# LEADERSHIP EXCELLENCE AND ADVANCEMENT OF DIRECTORS (LEAD) PROGRAM: FIRST INSIGHTS FROM A NEW MENTORSHIP MODEL

**DOI:** 10.1093/geroni/igae098.3863

**Published:** 2024-12-31

**Authors:** Juanita Bacsu, Harleah Buck

**Affiliations:** Thompson Rivers University, Kamloops, BC Canada, British Columbia, Canada; University of Iowa, Iowa city, Iowa, United States

## Abstract

Mentorship is critical to advancing leadership skills and building confidence in academia. However, there is a paucity of programs focused on supporting new center directors in the field of aging. Working with the Gerontological Society of America, we created the Leadership Excellence and Advancement of Directors (LEAD) Program as a mentorship model to provide peer support, networking opportunities, professional growth, and skill development for new center directors. This presentation aims to: 1) identify critical planning steps and strategies to develop high quality mentorship programs to support new center directors in the field of aging; and 2) recognize the LEAD Program as an emerging prototype to elevate leadership skills, build confidence, and advance research excellence to support new directors of gerontological research centers. The Lead Program is a groundbreaking GSA model that is geared towards new directors by providing peer support, leadership development, mentorship, and a dynamic network of fellow directors. The need for the LEAD Program was identified during a recent GSA Center Directors meeting where several directors shared that they were retiring and new directors were looking for mentorship support. The development of the LEAD Program was cultivated by: i) identifying priority topic areas and needs; ii) establishing meeting forma and frequency; iii) addressing potential program barriers and obstacles; and iv) exploring opportunities for peer support and collaboration to advance new directors in the field of aging. The next steps are to implement and evaluate the LEAD Program to strengthen the mentorship model for future cohorts of center directors.

